# A Systems Approach to Identify Factors Influencing Participation in Two Tribally-Administered WIC Programs

**DOI:** 10.3390/nu15051210

**Published:** 2023-02-28

**Authors:** Michelle Estradé, Samantha Grace Alarcon Basurto, Abbegayle McCarter, Joel Gittelsohn, Takeru Igusa, Siyao Zhu, Lisa Poirier, Susan Gross, Marla Pardilla, Martha Rojo, Kevin Lombard, Henry Haskie, Veronica Clark, Jacqueline Swartz, Yeeli Mui

**Affiliations:** 1 Johns Hopkins Bloomberg School of Public Health, 615 N Wolfe St., Baltimore, MD 21205, USA; 2 Whiting School of Engineering, Johns Hopkins University, 3400 North Charles St., Baltimore, MD 21218, USA; 3 College of Nursing, University of Arkansas for Medical Sciences, 220 UAMS Campus Dr., Little Rock, AR 72205, USA; 4 College of Argicultural, Consumer, and Environmental Sciences, New Mexico State University, 300 Road 4063, Farmington, NM 87401, USA; 5 Navajo Nation WIC, Window Rock, NM 86515, USA

**Keywords:** WIC, Native American, system dynamics, causal loop diagram, health equity, nutrition

## Abstract

Native American populations experience highly disproportionate rates of poor maternal-child health outcomes. The WIC program aims to safeguard health by providing greater access to nutritious foods, but for reasons not well understood, participation in many tribally-administered WIC programs has declined to a greater extent compared to the national average decline in participation over the last decade. This study aims to examine influences on WIC participation from a systems perspective in two tribally-administered WIC programs. In-depth interviews were conducted with WIC-eligible individuals, WIC staff, tribal administrators, and store owners. Interview transcripts underwent qualitative coding, followed by identifying causal relationships between codes and iterative refining of relationships using Kumu. Two community-specific causal loop diagrams (CLDs) were developed and compared. Findings from interviews in the Midwest yielded a total of 22 factors connected through 5 feedback loops, and in the Southwest a total of 26 factors connected through 7 feedback loops, resulting in three overlapping themes: Reservation and Food Store Infrastructure, WIC Staff Interactions and Integration with the Community, and State-level Administration and Bureaucracy. This study demonstrates the value of a systems approach to explore interconnected barriers and facilitators that can inform future strategies and mitigate declines in WIC participation.

## 1. Introduction

Native Americans have endured centuries of genocidal actions and policies, including the intentional destruction of traditional foodways and livelihoods, that have resulted in an inequitable burden of poverty and chronic disease [1,2]. Indigenous maternal-child health disparities can be further contextualized by systemic factors rooted in the social determinants of health, such as difficult access to insurance and health care, discrimination, and a mistrust in health care providers as a result of historical trauma [3]. Native American women are three to four times more likely to die of complications related to pregnancy and childbirth compared to white women in the U.S. [4], and infant mortality in this group is 70% higher than among non-Hispanic whites [5].

Participation in the Special Supplemental Nutrition Program for Women, Infants, and Children (WIC) is associated with improved maternal health outcomes, improved birth outcomes, better nutrition status, food security, and better child health [6,7,8,9,10], all of which have implications for health equity. While WIC participation at a national level declined by approximately 21% from 2014–2021 [11], participation has declined more steeply in many tribally-administered WIC programs for reasons not yet understood. For example, the Navajo Nation and Keweenaw Bay Indian Community (KBIC) WIC programs, with whom we partnered for this study, experienced over 50% declines in participation from 2014–2021 [11].

Previous studies have identified multiple challenges to WIC participation, including poor labeling of WIC-approved foods, frustration with the variety and availability of WIC-approved items in local stores, social stigma, and confusion about WIC eligibility criteria [12]. Structural barriers such as access to transportation and scheduling conflicts also impact WIC participation and disproportionately affect marginalized populations in rural areas [13].

A promising approach to understanding the complex influences on WIC participation is using systems science [14]. In particular, community-based system dynamics is a participatory modeling approach that involves building mental maps, or causal loop diagrams (CLDs), to examine system behaviors. Through group model building (GMB), which is a highly interactive and iterative process, key stakeholders with deep understanding and lived experiences develop and refine conceptual models of complex public health problems [15,16]. 

Causal loop diagrams have been used to examine urban food systems [16] and to explore intervention strategies for chronic disease management [17], but this methodology has not yet been leveraged to elucidate the problem of declining participation in tribally-administered WIC programs. Therefore, this work aims to explore the following research question by applying community-based system dynamics: What are the interconnected barriers and facilitators influencing participation in two tribally-administered WIC programs?

## 2. Materials and Methods

We selected two sites for this study, representing one small and one large tribally-administered WIC program. These were both sites in which tribal members expressed an interest to explore reasons for declining participation in their WIC programs and where the project team and principal investigator had worked previously over a period of 10–20 years.

The original study plan included recruitment of key stakeholders to participate in a group model building workshop to produce a causal loop diagram (CLD), representing the community-level system of factors impacting WIC participation. However, pandemic-related restrictions and connectivity challenges in rural reservation communities required a shift to alternate methods. Instead of group model building workshops held in person, we remotely conducted key stakeholder interviews to explore reasons for declining WIC participation and utilized a method described by Kim & Andersen [18] to extract information from interview transcripts that was used in building the CLDs (see further details below).

Prior to commencing with recruitment, the study protocol was reviewed and approved by the Johns Hopkins Bloomberg School of Public Health Institutional Review Board, the Navajo Nation Human Research Review Board, and the National Indian Health Service Institutional Review Board.

### 2.1. Setting

#### 2.1.1. WIC Program in the Midwest U.S.

The tribally-administered Keweenaw Bay Indian Community (KBIC) WIC program operates as a local agency within the jurisdiction of the Michigan state WIC agency in the northern-central part (i.e., Midwest) of the U.S. The reservation spans approximately 100 mi^2^ (259 km^2^), with over 1100 tribal members living on reservation lands. In fiscal year 2021, the program served 51 women and 135 infants and children [19]. There are two small grocery stores on the reservation that accept WIC benefits, and larger supermarkets are located off the reservation, 35–60 (56–97 km) miles away. The WIC clinic at this site is located within the tribal health clinic building and employs one full-time staff (considered a single-certifier site), as well as a breastfeeding peer counselor. A registered dietitian is also contracted as a consultant and provides services as needed.

#### 2.1.2. WIC Program in the Southwest U.S.

Relative to the KIBC WIC program, Navajo Nation WIC operates on a larger scale as one of 33 Indian Tribal Organizations (ITO) with the same level of authority as a state WIC agency. With territory in the southwestern states of New Mexico, Arizona, and Utah, the Navajo Nation reservation covers approximately 27,000 mi^2^ (70,000 km^2^) with over 173,000 tribal members living on reservation lands. Participants are served at 12 clinic-based offices across the reservation, with 14 additional remote sites located inside of community centers. In fiscal year 2021, the program served 804 women and 3805 infants and children [20]. There are at least 70 vendors that accept WIC benefits on the reservation, including 13 grocery stores.

### 2.2. Recruitment

Approximately 15 key stakeholders in each community were recruited for in-depth qualitative interviews (*n* = 31), including WIC-eligible individuals, WIC staff, tribal administrators, and store owners. Current and former WIC participants were recruited via flyers posted in public locations such as grocery stores, gas stations, restaurants, laundromats, and the local tribal health clinic. The same flyers were also shared on relevant Facebook pages with consent from local partners, including community centers, WIC clinics, and Head Start programs. Recruitment flyers were sent via email to WIC staff and tribal health administrators in both communities. Food vendors were recruited via telephone. Recruitment was stratified by participant type, with a goal of obtaining at least 2 interview participants in each category. Table 1 shows the final number of interviews collected by stakeholder category and location.

### 2.3. Data Collection

Semi-structured in-depth interviews covered topics related to WIC enrollment and access, staff-client interactions, stocking of WIC foods in stores, acceptability of the WIC food package, and use of WIC benefits. Trained interviewers used an interview guide containing open-ended prompts such as “Please describe a recent food shopping trip when you used your WIC benefits.” Interviews were conducted between September 2021 and June 2022 via telephone, Zoom, or in person, depending on pandemic-related restrictions and participant preference. Interviews lasted approximately one hour and were either digitally recorded and later transcribed or underwent live transcription without voice recording. Verbal consent was confirmed both at the time of recruitment and immediately prior to each interview, and participants were sent a Visa gift card upon completing their interview.

### 2.4. Data Analysis

Eight interview transcripts underwent independent inductive open coding by three research team members in order to develop an initial codebook. The research team met multiple times during the open coding process to discuss codes and iteratively refine the codebook.

Following the methods described by Kim & Andersen [18], coded sections of text were used to generate cause-and-effect statements and populate a cause-and-effect factors chart describing direction of influence and relationship polarity between factors. Using Kumu [21], two comprehensive causal loop diagrams (CLDs) were developed (one for each community), representing barriers and facilitators to WIC participation. The CLDs underwent iterative refinements by the research team to create feedback loops from the linear word-and-arrow diagrams that emerged from the charting stage. When there were common factors in a pair of feedback loops, the loops were joined at these factors. Feedback loops were then categorized into broad descriptive themes. Across both CLDs, each feedback loop was discussed among the research team to decide whether it fit within one of the current themes or warranted creation of a new theme. A videoconference was then held with WIC directors and staff to present an early version of the CLD and elicit feedback in order to make refinements.

## 3. Results

**Figure 1:**
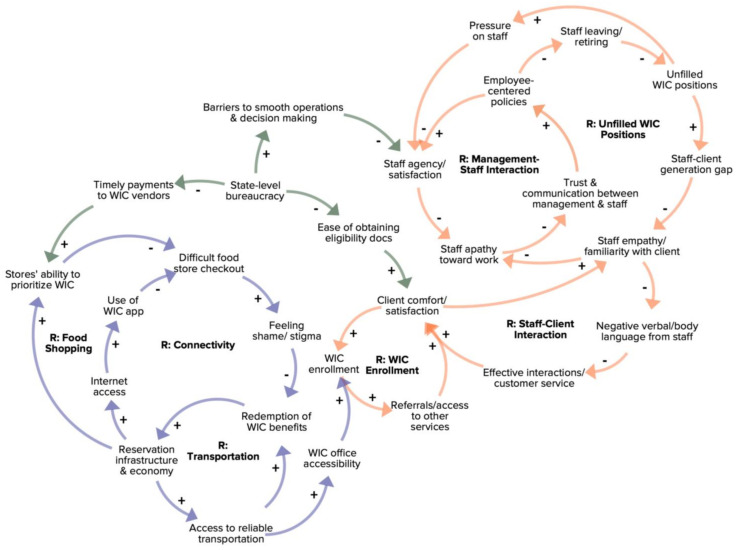
Causal loop diagram of factors influencing participation the Navajo Nation WIC program. R: Reinforcing Loop; green arrow: State-level Administration and Bureaucracy theme; purple arrow: Reservation and Food Store Infrastructure theme; orange arrow: WIC Staff Interactions and Integration with the Community theme. +: connected factors change in the same direction; −: connected factors change in opposite directions.

**Figure 2:**
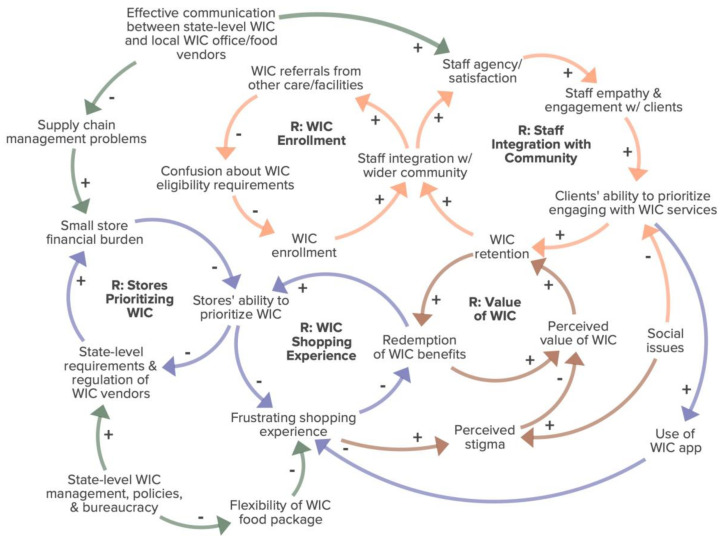
Causal loop diagram of factors influencing participation in the KBIC WIC program. R: Reinforcing Loop; green arrow: State-level Administration and Bureaucracy theme; purple arrow: Reservation and Food Store Infrastructure theme; orange arrow: WIC Staff Interactions and Integration with the Community theme; brown arrow: Perceived Value of WIC theme. +: connected factors change in the same direction; −: connected factors change in opposite directions.

In-depth interviews in KBIC yielded a total of 22 factors connected through 5 feedback loops, and in Navajo Nation a total of 26 factors connected through 7 feedback loops (Figure 1 and Figure 2). The two CLDs have three broadly-overlapping themes in common: Reservation and Food Store Infrastructure (purple arrows), WIC Staff Interactions and Integration with the Community (orange arrows), and State-level Administration and Bureaucracy (green arrows). The KBIC CLD includes one additional theme entitled Perceived Value of WIC (brown arrows).

### 3.1. Theme 1: Reservation and Food Store Infrastructure

In the Navajo Nation context, reservation infrastructure presented numerous barriers to WIC participation. Low access to transportation (i.e., to travel vast distances) made it difficult for participants to get to WIC food vendors and WIC clinics. Sparse telephone and internet connectivity were additional barriers when in-person service requirements were waived during the pandemic, as participants did not have a reliable way to contact WIC staff via phone or send in eligibility documents via e-mail. The Connectivity reinforcing loop in Figure 1 depicts how limitations in reservation infrastructure and economy lead to low internet access, which reduces clients’ ability to use a WIC cellphone application meant to improve the shopping experience by identifying client-specific WIC-eligible food items.

Though numerous WIC clients in Navajo Nation reported receiving a free government-subsidized phone through the CellularOne Tribal Lifeline program, connectivity in some areas of the reservation remains a barrier to use of the phones. As a consequence, the checkout process at food stores remains stigmatizing which continues to disincentivize the redemption of WIC benefits, thereby limiting economic activity by decreasing food stores’ potential sales. One WIC participant described:

“*[The free phone from Cellular One] is pretty good. I didn’t have any trouble because I go here and there with my children for our appointments, and even in border towns I was able to still get phone calls and messages. But not sure how the internet would work [in rural areas]. I have to connect to WiFi to get internet. I don’t have it on the phone.*”

In KBIC, food retailer infrastructure was described as a barrier to WIC benefit redemption. In Figure 2, the reinforcing loop entitled Stores Prioritizing WIC depicts how state-imposed requirements and regulation of WIC vendors increases the financial burden on small stores, which in turn decreases the stores’ ability to prioritize WIC through cashier training, stocking WIC foods, clearly marking WIC-eligible foods, and keeping cash register systems updated. As stores’ ability to prioritize WIC decreases, this increases the state’s perceived need to enforce regulations and penalties for non-adherence to WIC vendor guidelines, further reinforcing the financial burden on small stores. As one store manager shared:

“*The WIC items have to [be scanned] on the little WIC EBT machine, and the other items are going to come through the register. I wish we could update our register system and do everything on the register, but money doesn’t allow that right now.*”

In both Navajo Nation and KBIC, as stores’ ability to prioritize WIC decreases, people shopping with WIC benefits report more frustrating shopping experiences, which disincentivize the redemption of WIC benefits. If WIC benefit redemption decreases, stores priorities around WIC product stocking, signage, and cashier training decrease, reinforcing a frustrating shopping experience for WIC participants. In contrast, one WIC participant gave an example of a small store that created a positive shopping experience by prioritizing WIC:

“*A little local store here, they have a WIC shelf. Everything on that shelf is for WIC—it’s perfect. That works, and I praise them for that.*”

### 3.2. Theme 2: WIC Staff Interactions and Integration with the Community

The KBIC WIC program’s integration with the wider community was identified as a positive and important aspect of the success of the WIC program (Figure 2). For example, the reinforcing loop entitled Staff Integration with Community shows that as WIC staff integration with the wider community increases, staff agency and satisfaction increase. More staff agency and satisfaction lead to higher empathy and engagement with clients, which in turn increases clients’ability to prioritize engaging with WIC services. As engagement with WIC services increases, WIC retention increases and reinforces WIC staff’s integration with the community. As one tribal health administrator explained,

“*…we have a really good staff and I know that our tribe in particular, in our health center, you know we’re very resilient no matter what happens… I’m not doing it by myself; I have this great group of people to work with.*”

In Navajo Nation, WIC staff-client interactions were described as a key factor impacting participant satisfaction. The reinforcing loop entitled Staff-Client Interactions shows that when staff empathy and familiarity with WIC clients decreases, clients’ reports of negative verbal and body language from staff increases, which decreases the effectiveness of their interactions and client satisfaction. In particular, communication issues around paperwork requirements to maintain WIC eligibility are frequently cited as a source of confusion and frustration for clients and staff. As client comfort and satisfaction decreases, staff empathy and familiarity with WIC clients is more likely to be negatively affected and reinforced. In addition, WIC enrollment decreases and as clients try to distance themselves from staff to reduce tension, this leads to fewer referrals that help clients access other health and assistance services, further reinforcing decreased client comfort and satisfaction. As one WIC participant explained,

“*…their customer service, I’m not a big fan of it. I would be nice and cooperative and listen to just hurry up and get out of there.*”

Staff empathy and familiarity with clients was also a key factor contributing to two interconnected feedback loops entitled WIC Management-Staff Interaction and Unfilled WIC Positions.

### 3.3. Theme 3: State-level Administration and Bureaucracy

This theme relates to exogenous factors that were found to be common across communities, contributing to feedback loops in both systems. In Navajo Nation, bureaucracy at the tribal nation level created barriers for WIC participants to obtain necessary documents to prove WIC eligibility, as well as barriers to localized decision making in WIC clinics. One WIC participant illustrated the point with this example:

“*…even just like getting an ID, getting a driver’s license, all of those things are so much more difficult because I live on the Navajo Nation. I mean, I’m 27 now and I still don’t have my driver’s license because it’s so difficult to go through the bureaucratic process.*”

In KBIC, bureaucracy at the state-level impacted WIC administration by hindering effective communication with the local WIC clinic and local food vendors, creating supply chain issues and decreasing local clinic staff agency and satisfaction. A store manager gave one example of the problem:

“*Our [WIC EBT reader] machine went down when they did an update, and when we called the state to find out about it, they said ‘well you know you’re not set up with the new machines’…and it’s like, really? Why didn’t you give us a heads up and get these new WIC machines in here? So we ended up going, it was a good two weeks, without WIC…*”

Though it did not emerge as a major component in the CLDs, the cultural beliefs and values shared by WIC staff and participants were mentioned in both study locations as a value-added aspect of WIC services. In some cases, however, they were limited by WIC’s requirement to utilize only U.S. federal (western evidence-based) recommendations, which created a barrier to sharing traditional knowledge around maternal-child health. As of the WIC staff explained:

“*With WIC I have to stay within their guidelines, you know, and culturally… I can’t give [participants] that culture. Because technically I gotta stay within my scope. So that’s where I have a hard time with WIC.*”

### 3.4. Theme 4: Value of WIC

The perceived value of WIC was a theme that emerged from interviews in KBIC and resulted in a reinforcing feedback loop of the same name. As the perceived value of WIC increases, participant retention in the WIC program increases. The longer participants are retained in WIC, the more WIC benefits they will redeem over time, thereby reinforcing their perceived value of WIC. One WIC participant shared,

“*It’s really hard to budget. Because I get paid every two weeks, um, when it gets close to my next paycheck I look at my account and I have maybe $20 left. If I had to buy the formula, straight up, I don’t know what I’d do [without WIC].*”

The perceived value of WIC is also negatively impacted by perceived stigma associated with food assistance participation, which was an issue that came up in both Navajo Nation and KBIC. The feelings of stigma are caused by frustrating shopping experiences and by the presence of other stigmatizing social issues which decrease clients’ ability to prioritize engaging with WIC services. A former WIC participant shared:

“ *The local gas station used to accept [WIC], except if there was a line, you’d be put in the back of the line to just let people who were getting gas or getting this or that go in front, and then they would process you last. So that really sucked, and people complained, and now they don’t accept WIC, which also sucks because it was the closest store to me.*”

## 4. Discussion

This is the first study to utilize system dynamics to explore influences on participation in tribally-administered WIC programs. The resulting CLDs provide a more holistic, system-level view of WIC participation than would be possible by examining any single stakeholder group or theme individually. Overall, our findings align with previous studies in other populations and settings, showing that WIC participation is impacted by a difficult shopping experience, WIC clinic and food store accessibility, and by the perception of stigma around food assistance program participation [13,22,23]. However, this study provides added context and detail around the system-level realities of WIC participation in two rural reservation communities.

The main issues impacting WIC participation in both study locations were broadly related to infrastructure, bureaucracy, and effective communication. Though the two CLDs share overlapping themes, the dynamic factors involved in WIC participation vary greatly. In Navajo Nation, fundamental aspects of reservation infrastructure made WIC participation difficult, including phone and internet connectivity, as well as access to transportation. Lack of access to transportation has been frequently mentioned in the literature as a barrier to WIC participation because it limits client access to WIC offices and grocery stores [13,22,24,25,26]. Solutions that have been proposed to address this issue include the use of mobile WIC clinics [27] and online grocery shopping with WIC benefits [28]. Further, the COVID-19 pandemic context in our study showed how participation difficulties can be exacerbated if infrastructure is not accounted for. Very few studies have examined internet and phone connectivity as a factor in WIC participation. This will undoubtedly become a priority for future research as online grocery shopping and use of a WIC smartphone app to manage various aspects of WIC participation become more common [28]. In KBIC, food store infrastructure created complex downstream barriers, impacting stores’ ability to stock WIC items and accept WIC benefits. Ours is the first study to connect this issue with WIC participation and should be explored in more depth in future studies.

In both locations, state-level bureaucratic and administrative burdens created barriers to efficient operations in local WIC offices and food stores. This appeared, for example, in the form of difficulties obtaining and processing eligibility documents to enroll in WIC, and small food stores feeling heavily burdened by policies regulating WIC vendors. Previous studies have found that various aspects of bureaucracy, including means testing, compliance costs, and excessive paperwork, obstruct participation in assistance programs of all kinds and contribute to participant stigma [29,30]. Regulations and requirements around WIC food vendors vary greatly by state and have been acknowledged as a potential barrier to small stores accepting WIC benefits, which is particularly problematic in rural areas [31]. The overall impact of bureaucratic and administrative burdens on participation in the WIC program has not been fully elucidated and is an area for future research.

Lastly, effective communication as a key factor driving many feedback loops was mentioned by members of all stakeholder groups across both study sites. Empathy in WIC staff-client interactions was described as important for client satisfaction and retention. Likewise, transparency and trust were essential aspects of effective communication between WIC administrators and staff, and where those were lacking, a subsequent apathy towards work negatively impacted their interactions with clients. In addition, some of the staff-client interaction challenges described in this study may have been exacerbated by connectivity issues while in-person service was suspended during the COVID-19 pandemic. Previous studies have identified WIC staff-client interactions as integral to the effectiveness of the WIC program and in need of improvement in some areas [32], however there has been little mention of communication between WIC administrators and staff in the literature. Several strategies to improve the effectiveness of staff-client communication have included training WIC staff in client-centered nutrition education [33] and motivational interviewing [34]. Future work should explore strategies to improve communication between WIC administrators and staff and improve staff integration with the communities they serve.

An important contribution of this study is that it demonstrates the utility and feasibility of community-based system dynamics for building CLDs in rural Native American communities, which has not previously been done. The next step in this work would be to identify the most feasible points of capacity-building within each system by working with community stakeholders. For example, our CLDs suggest that in the Navajo Nation WIC program, levers that address infrastructural issues such as internet connectivity may help improve WIC access and participant satisfaction. In the KBIC program setting, levers that improve food stores’ ability to prioritize WIC, like State-level investments in store infrastructure (e.g., installing updated cash register systems), may be most effective for improving participation.

Understanding the systemic facilitators and barriers to food assistance program participation may also help reveal opportunities to move towards tribal food sovereignty. Low food access and affordability are major barriers to health in reservation communities [35,36,37], and food sovereignty movements are an important part of how Native communities address food access and restore wellbeing [38]. There are 574 federally-recognized tribes in the U.S., and although most tribes have reported that they would like to administer federal food assistance programs to their own citizens [39], only 33 ITOs operate WIC agencies. Our work may help by showing a promising way to more comprehensively strengthen and support tribally-administered WIC programs.

### Strengths and Limitations

Our study demonstrates the utility of systems thinking for exploring reasons for declining WIC participation and identifying intervention strategies. The advantage of using a systems thinking approach is that it focuses on the relationships among components within a system, as opposed to solely focusing on the components in isolation [40]. This is also one of the first uses of Kim & Andersen’s methodology to build a CLD based on in-depth stakeholder interviews. 

An important limitation of CLDs is that it only represents the perspectives of the informants who contributed to its creation, and therefore may be missing some important factors or relationships within the system. For example, as pandemic-related restrictions limited our ability to recruit or interview most participants in person, we were unable to obtain any food vendor interviews in Navajo Nation, and therefore this important perspective is missing from that CLD. We were also limited by the inability to hold a GMB workshop in person. Relying on interview data to build a CLD misses out on the community participatory nature of GMB. However, we aimed to address these limitations through many rounds of CLD iteration and member checking. Lastly, it is possible that further contextual factors unique to each study community might decrease the transferability of these findings for other communities and other settings.

## 5. Conclusions

The WIC program operates as part of a complex multilevel system, which few studies have examined. This study demonstrates the feasibility and utility of a system approach for identifying barriers to participation in tribally-administered WIC programs. Our work suggests that addressing communication issues (e.g., client-staff interactions, administrative delays, cultural factors, and stigma) and improving community infrastructure (e.g., internet connectivity, transportation, and small food store infrastructure) may have positive impacts on WIC enrollment and retention in these two communities.

## Figures and Tables

**Table 1:** In-depth interviews conducted, by stakeholder type and WIC program location.

Stakeholder Type	Navajo Nation	KBIC	Total
Long-time ^1^ WIC participants	4	3	7
New ^2^ WIC participants	1	4	5
Former WIC participants	3	1	4
WIC-eligible non-participants	1	2	3
WIC staff	5	2	7
Tribal health administrators	1	2	3
WIC food vendors	0	2	2
Total	15	16	31

^1^ ≥3 years of participation in WIC; ^2^ ≤1 year of participation in WIC.

## Data Availability

The data presented in this study are available on request from the corresponding author. The data are not publicly available due to respect for tribal data sovereignty.
